# Neurofunctional Abnormalities during Sustained Attention in Severe Childhood Abuse

**DOI:** 10.1371/journal.pone.0165547

**Published:** 2016-11-10

**Authors:** Lena Lim, Heledd Hart, Mitul A. Mehta, Andrew Simmons, Kah Mirza, Katya Rubia

**Affiliations:** 1 Department of Child & Adolescent Psychiatry, Institute of Psychiatry, Psychology & Neuroscience, King’s College London, London, United Kingdom; 2 Department of Neuroimaging, Institute of Psychiatry, Psychology & Neuroscience, King’s College London, London, United Kingdom; 3 NIHR Biomedical Research Centre at South London and Maudsley Foundation NHS Trust, London, United Kingdom; Birkbeck College, UNITED KINGDOM

## Abstract

Childhood maltreatment is associated with adverse affective and cognitive consequences including impaired emotion processing, inhibition and attention. However, the majority of functional magnetic resonance imaging (fMRI) studies in childhood maltreatment have examined emotion processing, while very few studies have tested the neurofunctional substrates of cognitive functions and none of attention. This study investigated the association between severe childhood abuse and fMRI brain activation during a parametric sustained attention task with a progressively increasing load of sustained attention in 21 medication-naïve, drug-free young people with a history of childhood abuse controlling for psychiatric comorbidities by including 19 psychiatric controls matched for psychiatric diagnoses, and 27 healthy controls. Behaviorally, the participants exposed to childhood abuse showed increased omission errors in the task which correlated positively trend-wise with the duration of their abuse. Neurofunctionally, the participants with a history of childhood abuse, but not the psychiatric controls, displayed significantly reduced activation relative to the healthy controls during the most challenging attention condition only in typical attention regions including left inferior and dorsolateral prefrontal cortex, insula and temporal areas. We therefore show for the first time that severe childhood abuse is associated with neurofunctional abnormalities in key ventral frontal-temporal sustained attention regions. The findings represent a first step towards the delineation of abuse-related neurofunctional abnormalities in sustained attention, which may help in the development of effective treatments for victims of childhood abuse.

## Introduction

There is increasing interest in understanding the effects of childhood adversities on the developing brain, given evidence that early environmental factors can have a substantial influence on the emerging brain architecture and long-term health of the person [1]. Childhood maltreatment, including physical, sexual and emotional abuse and neglect is common in the United Kingdom with pediatric prevalence rates of 7–10% [2]. Furthermore, childhood adversities are significantly associated with first onsets of various psychiatric disorders including mood, anxiety and post-traumatic stress disorders (PTSD) [3].

The psychopathological outcomes associated with childhood maltreatment may be mediated by the disruption of cognitive processes and their associated neural underpinnings [4]. Childhood maltreatment has been associated with adverse cognitive consequences such as low IQ and poor academic performance alongside impaired functions of motor and interference inhibition, sustained and selective attention, emotion and reward processing [5,6]. In particular, several neuropsychological studies have reported auditory [7,8] and visual [8–12] attention deficits in childhood maltreatment. Sustained attention, the ability to keep one’s mind continuously focused on a particular task, is a key dimension of attention control [13]. It is important for mature adult goal-directed behavior, thought to underpin “higher-level” attention processes such as selective and divided attention as well as general cognitive ability [14]. Children with maltreatment-related PTSD [9] and institutionalized children made more omission errors compared to healthy controls during sustained attention tasks, which was furthermore related to longer institutional care [15,16]. In adults, childhood physical abuse and neglect have also been associated with sustained attention deficits [17]. Additionally, population-based studies report significant associations between childhood maltreatment and Attention-Deficit/Hyperactivity Disorder (ADHD) like inattentive symptoms [18,19].

Despite the consistent neuropsychological findings of attention deficits in individuals with a history of childhood maltreatment, no fMRI study, to date, has examined sustained attention in this population The majority of fMRI studies in childhood maltreatment have examined brain function during emotion processing, given neuropsychological evidence of an attention bias and an increased sensitivity to angry and fearful expressions in maltreated children [20–23] and adults [24]. These studies found abnormally enhanced activation of fronto-limbic regions in response to negative emotions, in particular to angry and fearful facial expressions, in maltreated children/adolescents [25–28] and adults [29–31] relative to healthy controls, suggesting behavioral and neurofunctional hypersensitivity to fear and anger.

However, very few fMRI studies have tested cognitive processes known as “executive functions” which are particularly vulnerable to the deleterious effects of stress [32]. Executive functions include facets of high-order cognitions such as inhibition, working memory and sustained attention [33]. Using motor inhibition tasks, young people with a history of childhood maltreatment showed increased activation in inferior frontal cortex (IFC) and anterior cingulate cortex (ACC) during successful inhibition [34,35] and in typical dorsomedial prefrontal/ACC error-processing regions during failed inhibition [36]. In adults, a history of childhood maltreatment was associated with decreased functional connectivity of IFC and dorsal ACC during successful inhibition, which was furthermore related to impulsiveness and inattentive symptoms [37]. Using visual/verbal working memory tasks, childhood physical abuse was associated with reduced left cortical functioning in the frontal and temporal lobes in adults who were severely abused during childhood [38]. Also, higher levels of early childhood stress were associated with poorer spatial working memory in adolescents, and this relationship was mediated by smaller grey matter volume in the left prefrontal cortex (PFC) near the ACC and frontal poles [32].

Furthermore, a recent meta-analysis of structural MRI studies and reviews of both structural and functional MRI studies in childhood maltreatment show that individuals exposed to childhood maltreatment have deficits predominantly in two neuronal systems; one consisting of orbitofrontal-limbic circuits of top-down affect control, and another in the ventral attention system, in particular the ventral PFC, which is crucial for top-down cognitive control and sustained attention [6,39–41]. Hence, the findings suggest that the frontal-limbic regions are compromised both at the structural level and functional level during emotional processing in childhood maltreatment. On a similar vein, the findings that the (ventral) PFC is compromised anatomically [6,39] and functionally during executive functions in childhood abuse [34–36,38] and that structural deficits in the PFC mediated the relationship between childhood stress and poorer working memory [32] seem to suggest that that the ventral PFC may also be functionally impaired during sustained attention.

The aim of this study was therefore to test the hypothesis that medication-naïve, drug-free young people with a documented history of childhood physical abuse would exhibit activation deficits during sustained attention. Given that the fronto-parieto-temporal regions that mediate sustained attention develop relatively late in childhood and have been shown to be progressively more activated with increasing age between childhood and adulthood in fMRI studies [42,43], it is conceivable that childhood maltreatment interferes with the normal functional development of the ventral attention network. For this purpose, we used a parametrically modulated vigilance task requiring target detection with a progressively increasing load of sustained attention. Sexual abuse was excluded as it has been associated with different brain structure [44], behavioral and psychiatric consequences [45–51]. In particular, both childhood physical abuse and neglect, but not sexual abuse, were associated with alterations in regional corpus callosum size [49] and with grey matter reduction in a distributed corticostriatal-limbic system [52]. In terms of psychiatric and behavioral effects, childhood sexual but not physical abuse was associated with suicidal behaviour [47], self-injury [51], adulthood sexual dysfunction [53], and having at least two symptoms of schizophrenia [48]. Youth with childhood sexual abuse also reported more externalizing and internalizing problems over time than maltreated but non-sexually abused youth [46], and victims of childhood sexual abuse reported more severe mental pain compared with physical abuse victims [54]. Scholars have also argued that childhood sexual abuse is associated with experiences or feelings unique to sexual victimization relative to other abuse and neglect experiences; for example, traumatic sexualization, betrayal, stigmatization, attributions of responsibility as well as feelings of guilt and shame collectively may impact victims of childhood sexual abuse more profoundly and/or differently than victims of other abuse experiences [55,56]. For these reasons, and in order to obtain a more homogenous group, we only included children with physical abuse.

However, although the current study initially aimed to examine the neural correlates of sustained attention in young people exposed to physical abuse in childhood, it is unrealistic to separate physical abuse from typically co-occurring emotional abuse and neglect [57,58]. Claussen et al even noted that psychological maltreatment would be present in *almost all* cases of physical maltreatment. Hence, it is unlikely for the abused victim to experience *severe* physical abuse without experiencing at least moderate levels of emotional abuse and neglect concurrently; on the other hand, physical abuse does not always co-occur with sexual abuse.

Previous imaging studies in childhood maltreatment have several limitations such as not formally assessing and controlling for the presence of any co-occurring psychiatric conditions and/or substance abuse as well as the use of region of interest (ROI) analyses [6] which limits the search to a-priori hypothesized regions and provides a biased and inappropriately constrained characterization of anatomy or function [59]. In particular, given that large-scale epidemiological and longitudinal studies have consistently found that childhood maltreatment is linked developmentally to psychiatric disorders [3,60,61] and a meta-analysis study reported a causal relationship between non-sexual childhood maltreatment and a range of mental disorders [62], it is crucial to control for these in order to disentangle the effects of maltreatment from those associated with psychopathology [6,39–41]. Additionally, although some studies examined the neural correlates of childhood maltreatment in “healthy” participants [26–28,63], they relied solely on parental and/or participant’s self-ratings on clinical measures; hence, in the absence of a formal psychiatric assessment by a child psychiatrist, it remains unclear if these participants were without any psychiatric disorders at the point of testing. To assess the specificity of the association with childhood abuse, we therefore included a third group of psychiatric controls that was matched with the group with a history of childhood abuse on psychiatric comorbidities. Furthermore, to control for medication use or drug abuse, we included only participants who were drug-abuse free and medication-naïve. In addition, we conducted whole-brain analyses so that areas outside hypothesized regions would not be overlooked.

Therefore, we hypothesized that participants with a documented history of childhood physical abuse would show activation deficits in typical fronto-parieto-temporal sustained attention regions [64], in particular during the highest load of attention.

## Materials and Methods

### Participants

Seventy (23 young people with a history of childhood abuse, 20 psychiatric controls, and 27 healthy controls) right-handed, medication-naïve, drug-free and age-matched young people came to the laboratory for two sessions, and those below the age of 18 were accompanied by their guardians on both visits. In the first visit, demographic, clinical and abuse measures and IQ data were collected and the participants were acclimated to the scanner environment with an MRI replica. The fMRI sustained attention task was administered in the MRI scanner on the second visit. All participants were assessed by a child psychiatrist (KM) using the Development and Well-Being Assessment (DAWBA) [65] designed to generate ICD-10 and DSM-IV psychiatric diagnoses. The Strengths and Difficulties Questionnaires (SDQ) [66] and Beck’s Depression Inventory (BDI) [67] were used to provide psychopathology symptom scores. IQ was assessed using the Wechsler Abbreviated Scale of Intelligence (WASI) [68]. The Childhood Trauma Questionnaire (CTQ) [69] was used to measure the severity of childhood physical, emotional and sexual abuse as well as emotional and physical neglect. Socioeconomic status (SES) was measured by two non-sensitive items from the Family Affluence Scale (FAS) [70] on housing tenure and room occupancy.

Exclusion criteria for all participants were childhood sexual abuse, drug abuse, learning disability, neurological abnormalities, epilepsy, IQ < 70 and MRI contraindications. Urine screening for recent drug use was conducted with 10-panel urine drug test integrated cups (T-Cup; http://www.testfield.co.uk). All participants or their guardians if the participant was under the age of 18, provided written informed consent to participate in the study. Young people below the age of 18 were accompanied by their guardians during each visit and their guardians received the reimbursement for participating in the study on their behalf. The study was approved by the National Research Ethics Service Committee London-Fulham.

The 23 young people who experienced childhood physical abuse before the age of 12 were recruited through social services and psychiatric clinics. These young people, or their guardians if they were below the age of 18, were first asked to provide signed permission to contact their respective social services for written confirmation that there were official records of physical abuse. Next, the young people went on to complete the clinical interviews, abuse measures and IQ test and finally followed by the brain scan. The Childhood Experience of Care and Abuse (CECA) interview [71] was used to corroborate the CTQ and provide additional information on the abuse experience such as the age or onset and duration of abuse. All the participants with a history of childhood abuse scored ≥ 13 (i.e. the cut-off for severe/extreme physical abuse) [69] on the CTQ physical abuse subscale, and information from the CECA interview [71] and the CTQ were consistent with the official records. Psychiatric comorbidities included PTSD, depression, anxiety, conduct disorder and phobia. Two participants were excluded due to MRI motion artefacts, leaving a final sample of 21 participants (mean age 17.5, 15 male).

Twenty psychiatric controls were recruited through social services and psychiatric clinics. They had experienced no maltreatment (CTQ subscale scores of ≤ 7 for physical abuse, ≤ 8 for emotional abuse, ≤ 6 for sexual abuse, ≤ 9 for emotional neglect and ≤ 7 for physical neglect). The purpose of the inclusion of the psychiatric control group was to disentangle the effects due to maltreatment from those due to the psychiatric conditions that are typically associated with maltreatment. Therefore, diagnoses for both psychiatric controls and maltreated participants were made by the same experienced child psychiatrist (KM) using the same instrument, the DAWBA, and diagnoses were matched as closely as possible one-to-one with participants from the group with a history of childhood abuse. If there was any uncertainty regarding DAWBA diagnoses, Professor Robert Goodman, the psychiatrist responsible for the development of the DAWBA, checked and confirmed the diagnosis. The group with a history of childhood abuse and the psychiatric control group were well matched on psychiatric conditions (Table 1). Where psychiatric controls had PTSD, the causal trauma(s) were unrelated to childhood maltreatment and included bullying by peers, living in Afghanistan during the war, witnessing murder, experiencing a car accident and death of a loved one. One participant was excluded due to motion artefacts, leaving a final sample of 19 patients (mean age 16.9, 9 male).

**Table 1:** Demographic Characteristic of 21 Young People Exposed to Childhood Abuse, 19 Psychiatric Controls and 27 Healthy Controls.

	Childhood Abuse(N = 21)		Psychiatric Controls(N = 19)		Healthy Controls(N = 27)		Analysis		
	Mean	SD	Mean	SD	Mean	SD	F(2, 64)	*p* (corr.)	Group Comparisons
**Age (years)** [age range:13–20]	17.5	2.32	16.9	2.48	17.5	1.63	0.58	0.56	-
**Socioeconomic status**	2.77	0.69	2.94	0.66	3.22	0.75	2.43	0.10	-
**IQ**	90.0	12.6	93.6	13.0	105.4	10.1	11.3	0.001	CA, PC < HC
**Strengths and Difficulties Questionnaire:**									
*Emotional problems*	4.62	2.77	4.95	2.95	1.92	1.61	10.5	<0.001	CA, PC > HC
*Conduct problems*	4.43	2.01	2.37	2.36	1.68	1.60	11.5	<0.001	CA > PC, HC
*Hyperactivity*	5.38	2.40	4.68	2.65	2.84	2.14	7.08	0.002	CA, PC > HC
*Peer problems*	3.81	1.54	2.37	2.03	1.16	1.72	12.9	<0.001	CA > PC, HC
*Prosocial*	7.24	1.70	8.63	1.64	8.08	1.41	3.99	0.02	CA< PC
*Total difficulties score*	18.2	6.20	14.4	6.34	7.60	5.73	18.2	<0.001	CA, PC > HC
**Beck’s Depression Inventory**	16.0	10.6	21.1	12.1	5.92	6.09	8.32	< 0.001	CA, PC > HC
**Childhood Trauma Questionnaire:**									
*Physical abuse*	20.8	5.04	6.21	1.58	5.52	0.94	117.4	<0.001	CA > PC, HC
*Emotional abuse*	18.0	4.40	7.11	1.79	6.04	1.13	94.4	<0.001	CA > PC, HC
*Sexual abuse*	5.14	0.65	5.39	0.78	5.11	0.42	1.18	0.31	-
*Physical neglect*	14.0	5.02	6.74	2.26	5.59	1.22	36.9	<0.001	CA > PC, HC
*Emotional neglect*	18.3	3.93	8.79	3.69	7.93	3.35	50.7	<0.001	CA > PC, HC
**Age at onset of (physical)abuse (years)**	4.24	2.55							
**Duration of (physical) abuse(years)**	8.29	3.20							
	**N**	**%**	**N**	**%**	**N**	**%**	**χ**^**2**^	***p***	**Group Comparisons**
**Gender (Males)**	15	71	9	47	21	77	4.93	0.09	-
**Ethnicity:**							8.15	0.10	-
*Caucasian*	10	48	3	16	13	48			
*Afro-Caribbean*	8	38	10	52	12	44			
*Others (Asian/mixed)*	3	14	6	31	2	8			
**Psychiatric diagnosis:**									
*PTSD*	12	57	13	68	-				
*Depression*	6	29	6	31	-				
*Anxiety disorders*	4	19	5	26	-				
*Social phobia*	1	5	1	5	-				
*ADHD*	1	5	1	5					
*ODD/CD/Other disruptive behaviors*	4	19	3	16					

CA = childhood abuse; PC = psychiatric controls; HC = healthy controls; corr = Bonferroni corrected; CTQ = Childhood Trauma Questionnaire; ADHD = Attention Deficit Hyperactivity Disorder; PTSD = Post-Traumatic Stress Disorder; ODD = Oppositional Defiant Disorder; CD = Conduct Disorder

The 27 healthy controls (mean age 17.5, 21 male) with no history of psychiatric illness and childhood maltreatment (scoring below the same cut-offs as above) were recruited through advertisements in the same geographic areas of South London to ensure similar socioeconomic background (Table 1).

The methodology is similar to that reported in an earlier study [36] as the participants completed two additional fMRI tasks (stop and emotion processing), reported elsewhere [36].

### fMRI Paradigm: Sustained Attention Task (SAT)

**Fig 1:**
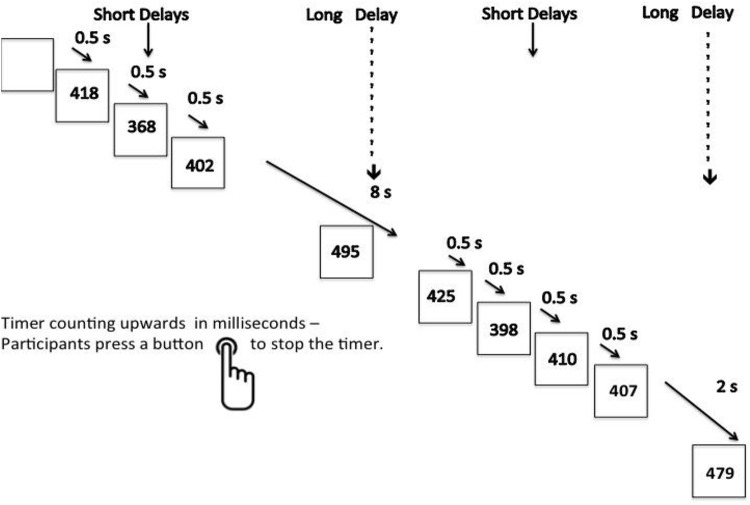
Schematic Representation of the Sustained Attention Task. Subjects are required to press a right-hand button as soon as they see a timer appear on the screen counting seconds. The counter appears after either predictable short delays of 0.5s in blocks of 3–5 stimuli, or after unpredictable long delays of 2s, 5s or 8s, pseudorandomly interspersed into the blocks of 0.5s delays. The long second delays have a progressively higher load on sustained attention than the short 0.5s delays that are typically anticipated and have a higher load on sensorimotor synchronization.

Participants practiced the task once prior to scanning. The 12-min SAT is a variant of psychomotor vigilance and delay tasks [42,72]. Participants need to respond as quickly as possible to the appearance of a visual timer counting up in milliseconds via a right hand button response within 1s. The visual stimuli appear either after short, predictable consecutive delays of 0.5s, in series of 3–5 stimuli (260 in total), or after unpredictable time delays of 2s, 5s or 8s (20 each), pseudo-randomly interspersed into the blocks of 3–5 0.5s delays. The long, infrequent, unpredictable delays place a higher load on sustained attention/vigilance while the short, predictable 0.5s delays are typically anticipated [73] placing a higher demand on sensorimotor synchronization [42,72,74] (Fig 1).

### Performance Data Analysis

Multiple repeated-measures analysis of variance (ANOVAs) with group as independent and delay as repeated measures were conducted to test for group (3 levels: Childhood abuse, Psychiatric controls, Healthy controls), delay (3 levels: 2s, 5s, 8s) and group by delay interaction effects using SPSS 18 on the following measures: mean reaction time (MRT), intra-subject variability of reaction time (SDintrasubject), omission and premature errors. A separate ANOVA for group differences for the short delays (0.5s) was also conducted on the same measures. Bonferroni adjustment was used for multiple comparisons.

### fMRI Image Acquisition

Gradient echo echo-planar MR imaging (EPI) data were acquired on a 3T GE Signa HDx system at the Centre for Neuroimaging Sciences, King’s College London. Stimulus images were projected on a screen, clearly visible through prism placed in front of participants’ eyes. The body coil was used for RF transmission and an 8-channel head coil for RF reception. During the 12-minute run of the task, in each of 28 non-contiguous planes parallel to the anterior-posterior commissural, 480 T_2_*-weighted MR images depicting Blood Oxygen Level Dependent (BOLD) contrast covering the whole brain were acquired with: echo time (TE) = 30ms, repetition time (TR) = 1.5s, 23 slices, flip angle = 70°, in-plane resolution = 3.75mm^2^, field of view (FOV) = 240mm, slice thickness/gap = 5/0.5mm, matrix = 64 x 64. A high-resolution gradient EPI was also acquired for accurate spatial normalization (TE = 30ms, TR = 3s, 43 slices, flip angle = 90°, in-plane resolution = 1.875mm^2^, FOV = 240mm, slice thickness/gap = 3/0.3mm, matrix = 128 x 128).

### fMRI Image Analysis

Image preprocessing and whole-brain analyses were carried out using Statistical Parametric Mapping software (SPM8, www.fil.ion.ucl.ac.uk/spm). Data were realigned to correct for subject movement and co-registered to the high-resolution gradient EPI, which was then used to estimate the parameters for spatially normalizing the data into a standard anatomical space (Montreal Neurological Institute). The resulting normalized volume time series was spatially smoothed using a Gaussian kernel of 8-mm full width at half maximum.

Data were analyzed within the framework of the General Linear Model. A single-subject (1^st^ level) model was created for each participant, including regressors encoding each experimental condition (long delays of 2s, 5s and 8s). The 0.5s short delay condition, which was not modelled and was used as an implicit baseline, was identical to the experimental conditions except for the delay period and hence controlled for visual stimulation and sensorimotor activation [42,72]. Each of these long delay conditions was contrasted with the 0.5s short delay condition that formed the implicit baseline. The model only examined correct trials; incorrect trials were omitted from the imaging analysis and included as covariates in the 1^st^ level model so that the variance due to errors can be accounted for and group differences in neural activity during the correct trials do not reflect any group differences in task performance. The movement parameters from the realignment procedure were also included as covariates in the 1^st^ level model. At the group-level, a whole-brain analysis was conducted using the SPM flexible factorial design model to examine the group by delay interaction effect, in which group was a between-subject factor (3 levels: Childhood abuse, Psychiatric controls, Healthy controls) and delay was a within-subject factor (3 levels: 2s, 5s, 8s). BOLD responses are reported using a family-wise error rate (FWE)-corrected cluster threshold of *p* < 0.05 and voxel threshold of *p* < 0.001. Next, mean BOLD contrast values for all voxels of significant clusters from the SPM flexible factorial analysis were extracted using MarsBaR (http://marsbar.sourceforge.net/) and a 3 (group) x 3 (delay) repeated-measures ANOVA followed by post-hoc *t*-tests (correcting for multiple comparisons) at each delay were conducted using SPSS 18 to determine between-group differences at each delay.

Finally, the significant clusters were also used to conduct exploratory correlation analyses with potential confounding variables such as IQ and age within each group, and with clinical and abuse measures (severity, age at onset and duration of abuse) within the group with a history of childhood abuse only.

## Results

### Subject Characteristics

The groups did not differ significantly in age, gender, ethnicity and SES but differed significantly in IQ, as expected (Table 1). Large-scale epidemiological studies have consistently reported an association between childhood maltreatment and lower IQ and cognitive abilities [75–77]. Therefore, since lower IQ is associated with childhood maltreatment, covarying for IQ when groups are not randomly selected and the covariate is a pre-existing group difference that did not occur by chance violates ANCOVA assumptions [78,79]. The primary data analyses are thus presented without covarying for IQ. However, to rule out any potential influence of IQ, a correlation analysis of IQ with brain activation in significant clusters and an additional confirmatory analysis on a subsample of IQ-matched participants were also conducted (see additional confirmatory analyses).

Although the study initially recruited participants with a history of childhood physical abuse, they also experienced marked/severe childhood emotional abuse and neglect (Table 1), which typically co-occur with physical abuse, and hence are a representative group of the childhood abuse population [57].

Healthy controls scored significantly lower on BDI (*p* < 0.01) and all SDQ difficulties subscales (*p* < 0.001) than the group with a history of childhood abuse, as well as on BDI (*p* < 0.001), SDQ emotional problems (*p* < 0.001) and hyperactivity (*p* < 0.05) subscales than psychiatric controls. The group with a history of childhood abuse scored significantly higher than psychiatric controls on SDQ conduct (*p* < 0.01) and peer problems (*p* < 0.05) but lower on prosocial (*p* < 0.01) subscales (Table 1).

### Task Performance

Paralleling the fMRI analyses where the 0.5s delay condition was included as an implicit baseline, and given that the long delay conditions (2s, 5s and 8s) were the targets of interest tapping into vigilance, we analyzed the long delay separately from the short delay conditions to assess effects of group, delay and group by delay interaction. There was no significant group effect on MRT (F (2, 64) = 1.99, *p* = 0.15) and SDintrasubject (F (2, 64) = 1.40, *p* = 0.26). There was a significant group effect on omission (F (2, 64) = 3.16, *p* < 0.05) and premature errors (F (2, 64) = 3.51, *p* < 0.05), due to the group with a history of childhood abuse and psychiatric control group making more omission errors than healthy controls and the group with a history of childhood abuse making more premature errors than healthy controls (Table 2). There were no significant effects of delay or of group by delay interaction (see Table in S1 Table for 0.5s delay results).

**Table 2:** Performance Measures for the Sustained Attention Task during 2s, 5s and 8s Delays for 21 Young People Exposed to Childhood Abuse, 19 Psychiatric Controls and 27 Healthy Controls.

		Childhood Abuse(N = 21)		Psychiatric Controls(N = 19)		Healthy Controls (N = 27)		Analysis				
	Delay	Mean	SD	Mean	SD	Mean	SD	Delay x Group F(2,64)	*p*(corr.)	Group F(2,64)	*p*(corr.)	Group Comparisons
Omission errors	2s	0.33	0.73	0.58	0.96	0.11	0.42	2.44	0.10	3.16	0.04	CA, PC >HC
	5s	0.57	0.93	0.37	0.60	0.19	0.48					
	8s	0.62	1.20	0.58	1.17	0.04	0.19					
Premature errors	2s	6.43	3.93	6.16	3.01	4.00	3.16	2.46	0.10	3.51	0.04	CA, PC >HC
	5s	7.38	4.65	6.84	3.39	4.30	3.74					
	8s	6.95	4.23	6.53	3.52	5.15	3.92					
MRT	2s	446	64	418	51	411	59	0.10	0.91	1.99	0.15	-
	5s	450	78	428	83	414						
	8s	449	87	420	65	408	80					
SDintrasubject	2s	101	50	71	31	74	38	1.82	0.17	1.40	0.26	-
	5s	93	50	74	46	85	61					
	8s	84	43	83	45	77	43					

MRT = mean reaction time (in ms); SDintrasubject = intrasubject variability of mean reaction times (in ms); corr = Bonferroni corrected; CA = childhood abuse; HC = healthy control; PC = psychiatric control

### Brain Activation

#### Motion

Multivariate ANOVAs showed no significant group differences in maximum translation (Wilks’ Lambda F (6,124) = 1.67, *p* > 0.05) or maximum rotation (Wilks’ Lambda F (6,124) = 1.09, *p* > 0.05) parameters.

#### Within Group Activations

**Fig 2:**
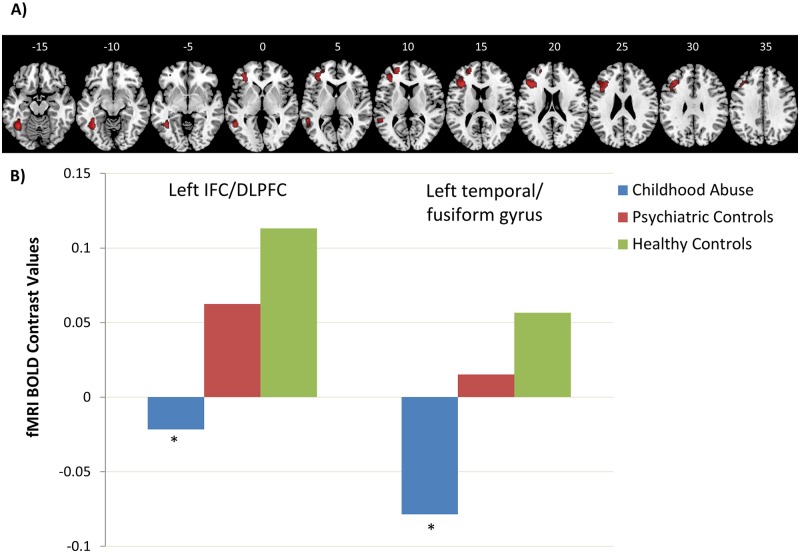
Group by Delay Interaction Effect on Brain Activation in Healthy Controls, Young People Exposed to Childhood Abuse and Psychiatric Controls. **A)** Axial sections showing group by delay interaction effect on brain activation during sustained attention in 27 healthy controls, 21 young people exposed to childhood abuse and 19 psychiatric controls as revealed by F test, *p* < 0.05 FWE-corrected at the cluster-level. Axial slices are marked with the z coordinate as distance in millimetres from the anterior–posterior commissure. The right side of the image corresponds to the right side of the brain. **B)** fMRI BOLD contrast values in Cluster 1 left IFC/DLPFC and Cluster 2 left temporal/fusiform gyrus for the healthy control group (green), childhood abuse group (blue) and psychiatric control group (red) during the 8s delay condition. The group with a history of childhood abuse had significantly lower activation in the left IFC/DLPFC cluster (p<0.05) and at a trend-level in the left temporal/fusiform cluster (p = 0.09) compared to heathy controls. There were no significant differences between the psychiatric and healthy controls.

Within-group activations for each of the 3 delays are shown in Fig 2 for each of the 3 groups. Briefly, healthy controls across the different delays had activation in a bilateral network comprising the SMA, paracentral lobule, middle and superior frontal gyri, superior temporal gyrus, insula, cingulate, post and precentral gyri, precuneus, cuneus, lingual, middle occipital gyrus, fusiform and cerebellum as well as in bilateral inferior frontal gyrus, middle temporal gyrus and striatum (S1 Fig). The group with a history of childhood abuse had activation in a bilateral network comprising the SMA, paracentral lobule, middle and superior frontal gyri, superior temporal gyrus, insula, cingulate, post and precentral gyri, precuneus, cuneus, lingual, middle occipital gyrus, fusiform and cerebellum (S2 Fig). The psychiatric controls had activation in bilateral SMA, paracentral lobule, post and precentral gyri, precuneus, cingulate, insula and superior temporal gyrus (S3 Fig).

#### Group Effect

When all 3 delays were considered together, there were no significant group differences across all delays (see S4 Fig for brain activations within each group and S5 Fig for the main effect of delay).

#### Group by Delay Interaction Effect

As hypothesized, there was, however, a significant group by delay interaction effect in two clusters: The first cluster comprised left IFC, anterior insula and dorsolateral prefrontal cortex (DLPFC) (*p* < 0.001). The second cluster comprised left inferior and middle temporal and fusiform areas (*p* < 0.001). Post-hoc analyses at each delay showed that the group with a history of childhood abuse had significantly reduced activation relative to healthy controls during the longest (8s) delay in the IFC cluster (*p* < 0.05) and at a trend-level in the temporal cluster (*p* = 0.09); however, they did not differ from psychiatric controls who did not differ from healthy controls (Table 3, Fig 2).

**Table 3:** Group by Delay Interaction Effect on Brain Activation between 21 Young People Exposed to Childhood Abuse, 19 Psychiatric Controls and 27 Healthy Controls.

Comparison and Brain Region	Brodmann’s Area	Cluster Level		Peak MNI Coordinates	Subject Contrast		
		No. of Voxels	*p*(corr.)		2s	5s	8s
***Cluster 1*:**							
Left orbitofrontal inferior/ middle frontal gyri/anterior insula	47/44/45/46/48/10/11/9/	969	<0.001	-38,26,16	-	**-**	CA<HC
				-24,52,12			
				-38,42,8			
***Cluster 2*:**							
Left inferior/middle temporal/ fusiform gyri	37/19/20/21/22	718	<0.001	-40,-54,-14	PC<CA	PC<HC	CA<HC^§^
				-54,-44,10			
				-40,-44,-10			

MNI = Montreal Neurological Institute; corr = FWE-corrected; CA = childhood abuse; PC = psychiatric controls; HC = healthy controls

^§^ Significant at trend-level p = 0.09

#### Correlation Analyses

To investigate whether the significant clusters of activation differences were associated with the main attention performance measure of omission errors and with clinical and abuse measures, BOLD values of each cluster for the 8s delay—with the greatest group differences—were extracted for each participant and correlated with omission errors at 8s within each group and with clinical and abuse measures (severity, age at onset and duration of abuse) within the group with a history of childhood abuse only.

For healthy controls, omission errors correlated negatively with activation in the IFC cluster (r = -0.69, *p* < 0.001) and temporal cluster (r = -0.55, *p* < 0.01). No significant correlations between omission errors and activation were observed in the group with a history of childhood abuse and the psychiatric control group. For the group with a history of childhood abuse, omission errors correlated at a trend-level positively with the duration of abuse (r = 0.4, *p* = 0.06). There were no significant associations between brain activation and age of onset/duration of abuse or SDQ symptoms scores in the group with a history of childhood abuse.

#### Additional (Confirmatory) Analyses

**Fig 3:**
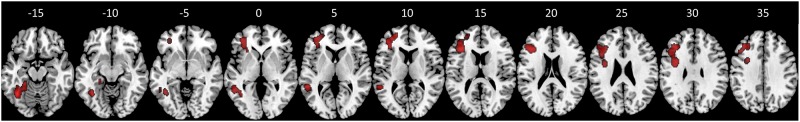
Group by Delay Interaction Effect on Brain Activation during Sustained Attention in an IQ-matched subsample of Healthy Controls, Young People Exposed to Childhood abuse and Psychiatric Controls. Axial sections showing group by delay interaction effect on brain activation during sustained attention in a subsample of 18 healthy controls, 19 young people exposed to childhood abuse and 19 psychiatric controls matched on IQ, as revealed by F test, *p* < 0.05 FWE-corrected at the cluster-level. Axial slices are marked with the z coordinate as distance in millimetres from the anterior–posterior commissure. The right side of the image corresponds to the right side of the brain.

Given that the group with a history of childhood abuse had significantly lower IQ than healthy controls, IQ was correlated with brain activation in the significant clusters and with the main performance measure of omission errors within each group. There were no significant correlations between IQ and brain activation or between IQ and omission errors (Table 4). Hence, higher brain activation or lesser omission error is not related to higher IQ and vice versa. Moreover, in order to further rule out the confounding effect of IQ on the findings, the analysis was repeated within a subgroup of IQ-matched sample (19 young people exposed to childhood abuse, 19 psychiatric controls and 18 healthy controls). All main findings remained significant in the IQ-matched subsample (Fig 3). Hence, IQ differences were unlikely to explain the findings.

**Table 4:** Correlations between Brain Activation, Task Performance, IQ and Age for 21 Young People Exposed to Childhood Abuse, 19 Psychiatric Controls and 27 Healthy Control^a^.

	**BOLD Response at 8s Delay**					
	**Cluster 1**			**Cluster 2**		
	**Healthy Controls**	**Childhood Abuse**	**Psychiatric Controls**	**Healthy Controls**	**Childhood Abuse**	**Psychiatric Controls**
**IQ**	0.09	0.12	0.22	0.31	0.09	0.28
**Age**	-0.02	0.09	0.46	-0.12	-0.11	0.41
	**Omission Error at 8s Delay**					
	**Healthy Controls**		**Childhood Abuse**		**Psychiatric Controls**	
**IQ**	-0.17		-0.08		-0.24	
**Age**	0.05		0.30		0.28	

^a^ All the Pearson correction coefficients are non-significant.

Furthermore, given that many individuals in the group with a history of childhood abuse suffered from PTSD, which was matched in the psychiatric control group, we wanted to explore whether PTSD alone was associated with potential brain activation abnormalities. For this purpose, we compared the 25 PTSD patients with the 27 healthy controls and with the 42 participants without PTSD. There were no significant group differences suggesting that the functional abnormalities are related to the abuse experience rather than PTSD.

Finally, we also controlled for variables such as gender, ethnicity and SES. The main findings remained significant when the extracted BOLD values of significant clusters were analyzed using repeated-measures ANCOVA in SPSS 18 covarying for these measures of gender, ethnicity and SES. Also, age did not correlate significantly with activation in the significant clusters or with omission errors within each group (Table 4). Thus, subtle and non-significant variations in these demographic factors were unlikely to have confounded the findings.

## Discussion

To our knowledge, this is the first fMRI study that examined the neurofunctional correlates of sustained attention in severe physical childhood abuse. We found that in a parametrically designed sustained attention task, medication-naïve and drug-free young people with a history of childhood physical abuse and concomitant neglect and emotion abuse relative to healthy controls exhibited increased omission errors, the main attention measure of the task, which furthermore was associated with a longer duration of abuse at a trend-level. At the neurofunctional level, only the group with a history of childhood abuse but not the psychiatric control group had reduced activation relative to healthy controls during the most challenging attention condition, the longest delay, in the ventral and dorsal frontal attention regions of left IFC, anterior insula and DLPFC as well as at a trend-level in middle and inferior temporal and fusiform areas.

Young people with a history of childhood physical abuse most of who also had neglect and emotional abuse showed reduced activation relative to healthy controls during the most challenging attention condition in left hemispheric ventral and dorsolateral prefrontal regions that are known to be important for sustained attention such as IFC/anterior insula and DLPFC [42,72,80–82]. The anterior insula is implicated in high-level cognitive control and attention processes [83] and together with IFC forms part of the ventral attention and salience network that facilitates the detection of important environmental stimuli, in this case the target stimuli [83,84]. The anterior insular/IFC network is thought to be involved in signaling the need for attentional effort to facilitate target detection, particularly under the more challenging condition of longer delays to prevent attentional drifts away from the task at hand [13,85]. The DLPFC plays a crucial role in top-down attention and is activated during visuospatial information processing and orienting of attention [86,87], including in this particular task version [42,72,88]. Finally, the temporal and inferior occipito-temporal regions are known to be involved in bottom-up visuospatial attention processes [89,90]. The implication of these fronto-temporal regions in sustained attention is furthermore reinforced in this study as they were negatively correlated with omission errors in healthy controls, suggesting that the higher the activation the better the task performance. Our findings therefore suggest that young people with a history of childhood abuse have a deficit in top-down IFC/DLPFC attention control and at a trend-level in bottom-up visuospatial attention processing.

The neurofunctional deficits during the longest delay may be abuse-related as they were not observed in the psychiatric control group, who did not differ from either the healthy control group or the group with a history of childhood abuse. This suggests that neurofunctional deficits in attention functions may be associated with abuse but also transfer to psychiatric complications. The findings of impairment in the most difficult condition only furthermore suggest that neurofunctional abnormalities during sustained attention in young people exposed to childhood abuse are intact in easier task conditions and manifest only during the most challenging condition. This is interesting in view of neurofunctional deficits in the same region of left DLPFC in this task in patients with autism spectrum disorder (ASD) and ADHD [42,72] during all delay conditions. It suggests less pervasive neurofunctional attention deficits in childhood abuse relative to other childhood disorders of attention, as they only manifested during the most challenging attention condition and were not observed throughout all task conditions.

The human brain is plastic and is continually modified by experience across development. Given that the IFC, DLPFC and temporal lobes are among the latest brain regions to develop structurally [91] and functionally [92], developing well into mid-adulthood, they may well be more susceptible to impairment following childhood adversity. In fact, our review [6] and a recent meta-analysis of structural MRI studies [36] in childhood abuse found grey matter abnormalities in the IFC, superior frontal and temporal regions. Furthermore, diffusion tensor imaging (DTI) studies in childhood maltreatment also reported abnormalities in left hemispheric fronto-temporal white matter tracts, including the uncinate fasciculus [93], arcuate fasciculus [94] as well as the inferior [95] and superior longitudinal fasciculus [96]. Hence, functional abnormalities in these late-developing IFC/DLPFC and temporal regions during sustained attention may suggest an environmentally triggered disturbance in the normal development of these attention networks as a consequence of childhood abuse.

At the performance level, the group with a history of childhood abuse made more omission errors than healthy controls, which was furthermore correlated with a longer duration of abuse. This is consistent with previous neuropsychological findings of more omission errors during sustained attention tasks in children with maltreatment-related PTSD [9] and in children with longer institutional care [15]. Furthermore, the frontal-temporal attention regions that were reduced in activation in the group with a history of childhood abuse during the longest delay condition were associated with less omission errors in healthy controls, suggesting that normally these regions are recruited for better performance while poor performance in the group with a history of childhood abuse may be due to poor recruitment of these regions.

The strength of this study is that all participants were medication-naïve, drug-free and that the physical abuse experience was carefully assessed and corroborated by social service records. Also, we included a psychiatric control group to determine the specificity of abuse. The inclusion of a “pure” group with a history of childhood physical abuse without any psychiatric disorders would have been a stronger control group to determine abuse-specific deficits; however, they would not be representative of the general childhood abuse populations as severe abuse is typically associated with psychiatric comorbidities [3,60–62]. Another limitation is that although the initial focus of the study was on childhood physical abuse, and sexual abuse was excluded as it has been shown to differ in many aspects [45], including distinctive effects on the somatosensory cortex [44], we cannot categorically state that the observed effects are a result of physical abuse exclusively as many participants also experienced neglect and emotional abuse. However, it is unrealistic to separate physical abuse from emotional abuse and neglect as the vast majority of maltreated children are subjected to more than one kind of abuse, with less than 5% of maltreatment occurring in isolation [97,98]. Moreover, using child protective services case records abstraction (physical, sexual, emotional abuse and neglect), latent class analysis revealed four distinctive profiles of childhood maltreatment experiences in which physical abuse was clustered with 1) neglect, 2) emotional abuse, 3) both neglect and emotional abuse and 4) neglect, emotional abuse and sexual abuse [98]. Thus, there is a high degree of overlap among childhood physical abuse, emotional abuse and neglect but the abused victim may not necessary had experienced sexual abuse. Nonetheless, it is worth noting the limitation in generalizing the current findings to individuals with a history of childhood sexual abuse.

The fact that IQ was not matched between the groups could be considered a limitation. However, since there was no significant correlation between IQ and brain activation in the two significant clusters and the findings remained in an additional confirmatory analysis on a subsample of IQ-matched participants, this suggests that IQ is unlikely to have confounded the findings. The lack of pubertal information is also a limitation given the significant development that occurs over this age range, particularly in the domain of executive functions. Also, it is unclear to what extent malnutrition, prenatal drug exposure and the presence of current life stressors may have influenced the findings. Finally, it is worth noting that comprehensive SES information has not been retrieved from parent/carer or social service report.

### Conclusions

In summary, we found that medication-naïve, drug-free young people with a history of childhood abuse, but not psychiatric controls, had functional activation deficits in typical sustained attention regions of IFC, DLPFC, insula and temporal areas compared to healthy controls during the longest and most challenging delay condition only. The findings represent a first step towards the delineation of abuse-related neurofunctional abnormalities in sustained attention, which may help in the development of effective treatments that target these regions.

## Supporting Information

S1 Fig Brain Activation for Healthy Controls during 2s, 5s and 8s Delays. Axial sections of activation during 2s, 5s and 8s delays for 27 healthy controls at FWE-corrected cluster-level threshold *p* < 0.05. Axial slices are marked with the z coordinate as distance in millimetres from the anterior–posterior commissure. The right side of the image corresponds to the right side of the brain.
(TIF)

S2 Fig Brain Activation for Young People Exposed to Childhood Abuse during 2s, 5s and 8s Delays. Axial sections of activation during 2s, 5s and 8s delays for 21 young people exposed to childhood abuse at FWE-corrected cluster-level threshold *p* < 0.05. Axial slices are marked with the z coordinate as distance in millimetres from the anterior–posterior commissure. The right side of the image corresponds to the right side of the brain.
(TIF)

S3 Fig Brain Activation for Psychiatric Controls during 2s, 5s and 8s Delays. Axial sections of activation during 2s, 5s and 8s delays for 19 psychiatric controls at FWE-corrected cluster-level threshold *p* < 0.05. Axial slices are marked with the z coordinate as distance in millimetres from the anterior–posterior commissure. The right side of the image corresponds to the right side of the brain.
(TIF)

S4 Fig Brain Activation across the 3 Delays for A) Healthy Controls, B) Young People Exposed to Childhood Abuse and C) Psychiatric Controls. Axial sections of activation during 2s, 5s and 8s delays for 27 healthy controls, 21 young people exposed to childhood abuse and 19 psychiatric controls at FWE-corrected cluster-level threshold *p* < 0.05. Axial slices are marked with the z coordinate as distance in millimetres from the anterior–posterior commissure. The right side of the image corresponds to the right side of the brain.
(TIF)

S5 Fig Main Effect of Delay on Brain Activation in Healthy Controls, Young People Exposed to Childhood Abuse and Psychiatric Controls. Axial sections showing main effect of delay on brain activation during sustained attention across 27 healthy controls, 21 young people exposed to childhood abuse and 19 psychiatric controls, as revealed by F test, *p* < 0.05 FWE-corrected at the cluster-level. Axial slices are marked with the z coordinate as distance in millimetres from the anterior–posterior commissure. The right side of the image corresponds to the right side of the brain.
(TIF)

S1 Table Performance Measures for the Sustained Attention Task during 0.5s Delay for 21 Young People Exposed to Childhood Abuse, 19 Psychiatric Controls and 27 Healthy Controls. (DOCX)
