# Supplementary figures and images for: Neurofunctional Abnormalities during Sustained Attention in Severe Childhood Abuse
Source: PLoS One. 2016 Nov 10;11(11):e0165547. doi: 10.1371/journal.pone.0165547 (PMC5104469; doi:10.1371/journal.pone.0165547)

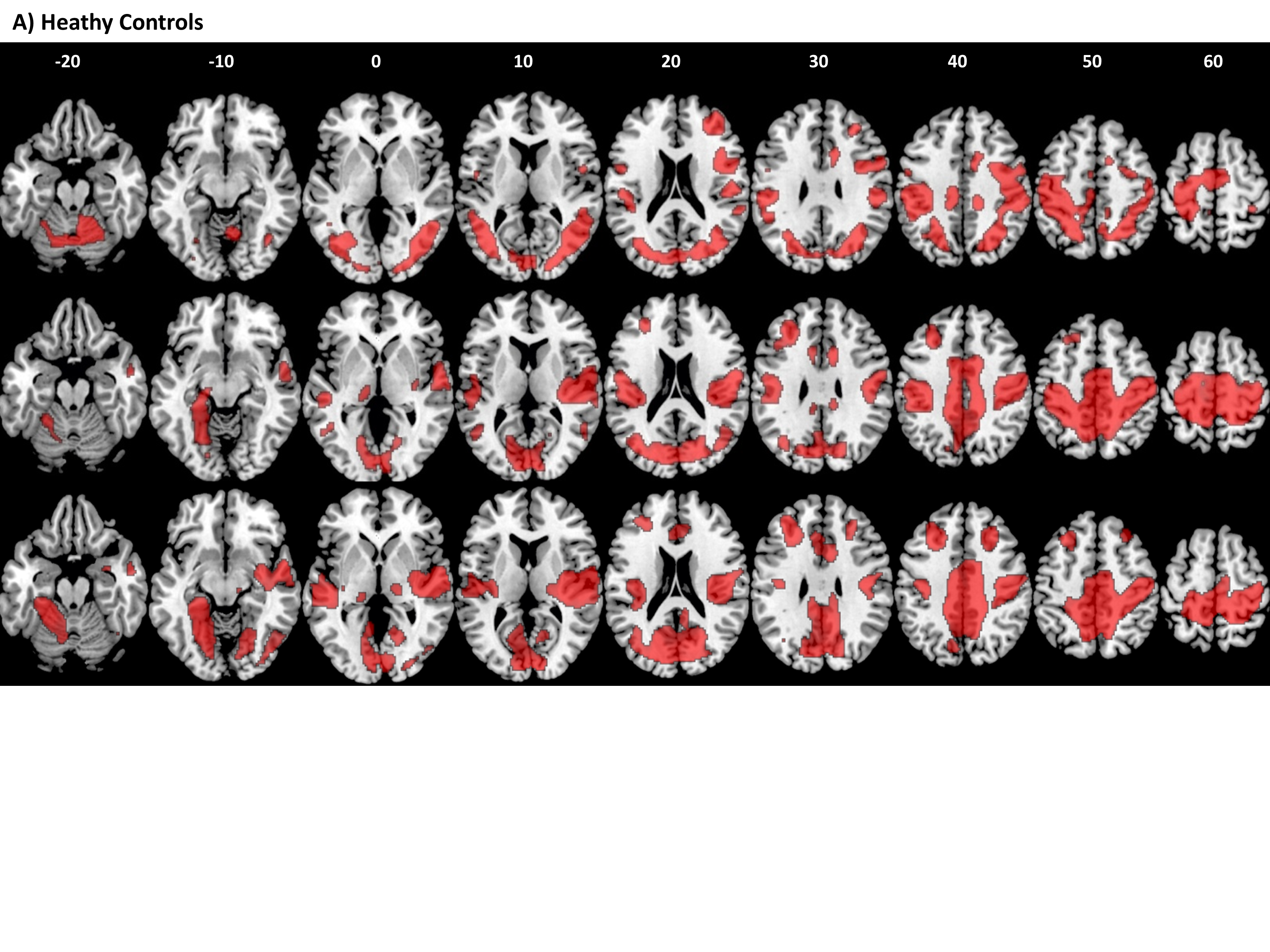

Supplement: S1 Fig — Axial sections of activation during 2s, 5s and 8s delays for 27 healthy controls at FWE-corrected cluster-level threshold p < 0.05. Axial slices are marked with the z coordinate as distance in millimetres from the anterior–posterior commissure. The right side of the image corresponds to the right side of the brain. (TIF) [file pone.0165547.s001.tif]

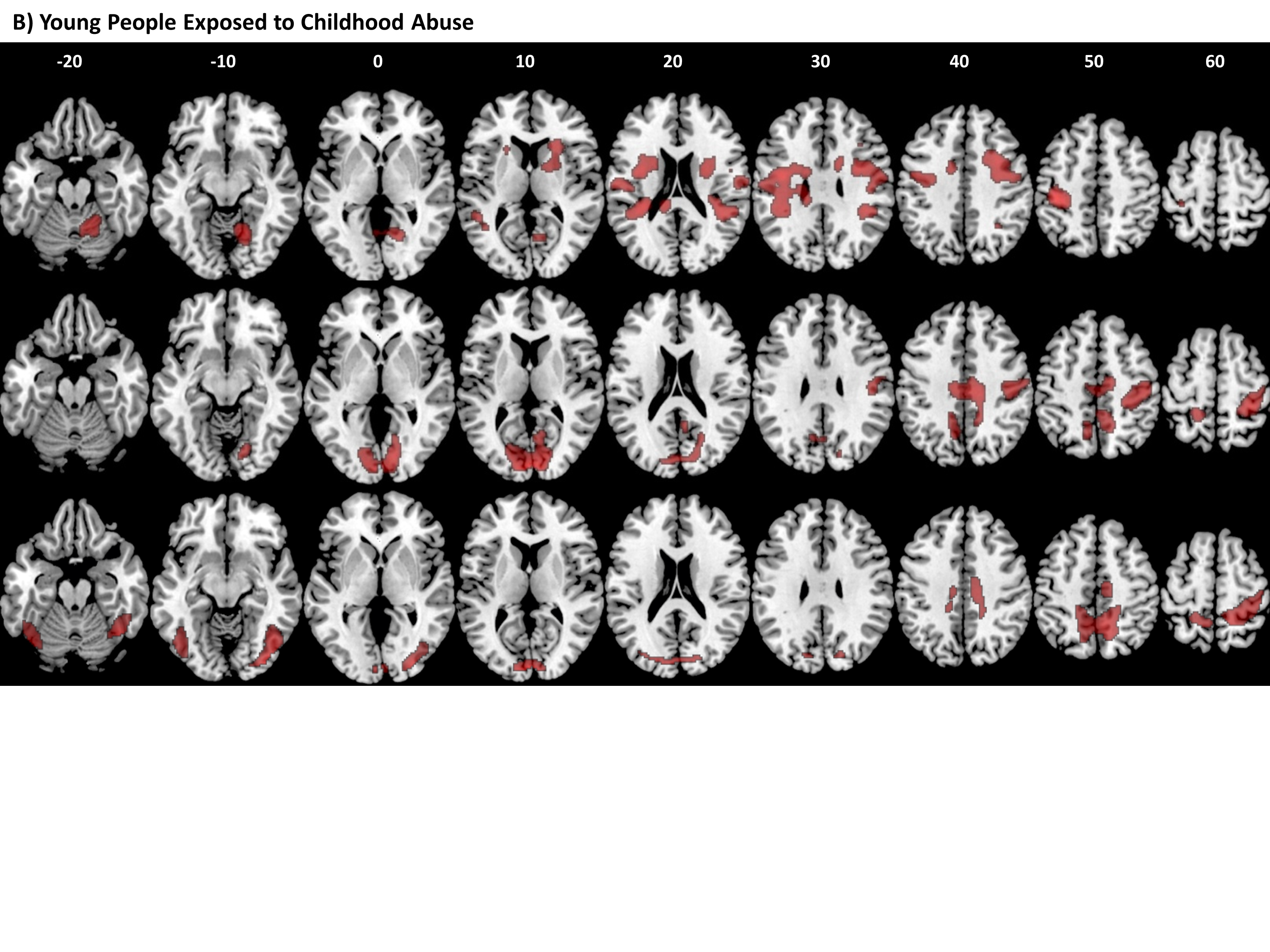

Supplement: S2 Fig — Axial sections of activation during 2s, 5s and 8s delays for 21 young people exposed to childhood abuse at FWE-corrected cluster-level threshold p < 0.05. Axial slices are marked with the z coordinate as distance in millimetres from the anterior–posterior commissure. The right side of the image corresponds to the right side of the brain. (TIF) [file pone.0165547.s002.tif]

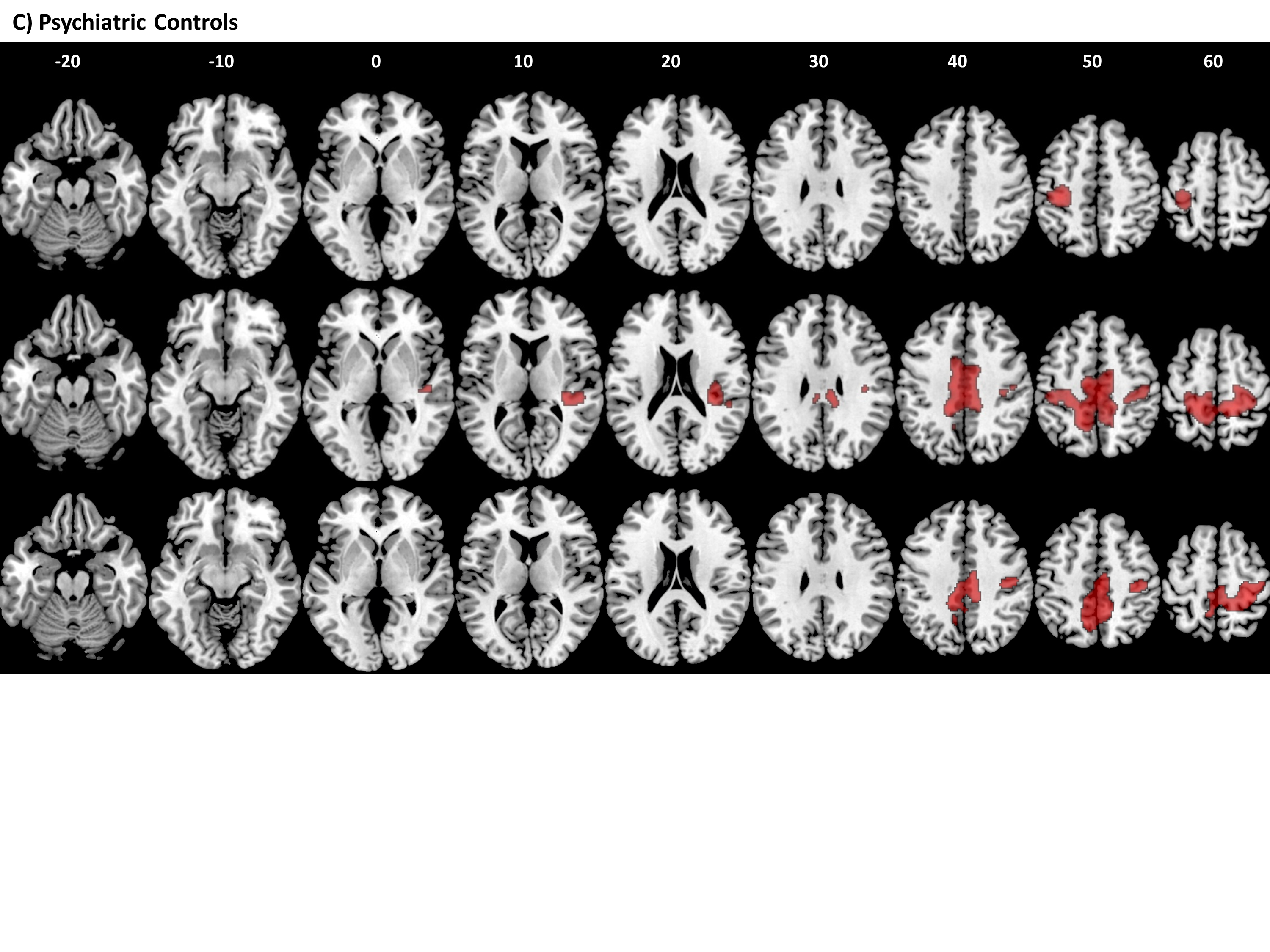

Supplement: S3 Fig — Axial sections of activation during 2s, 5s and 8s delays for 19 psychiatric controls at FWE-corrected cluster-level threshold p < 0.05. Axial slices are marked with the z coordinate as distance in millimetres from the anterior–posterior commissure. The right side of the image corresponds to the right side of the brain. (TIF) [file pone.0165547.s003.tif]

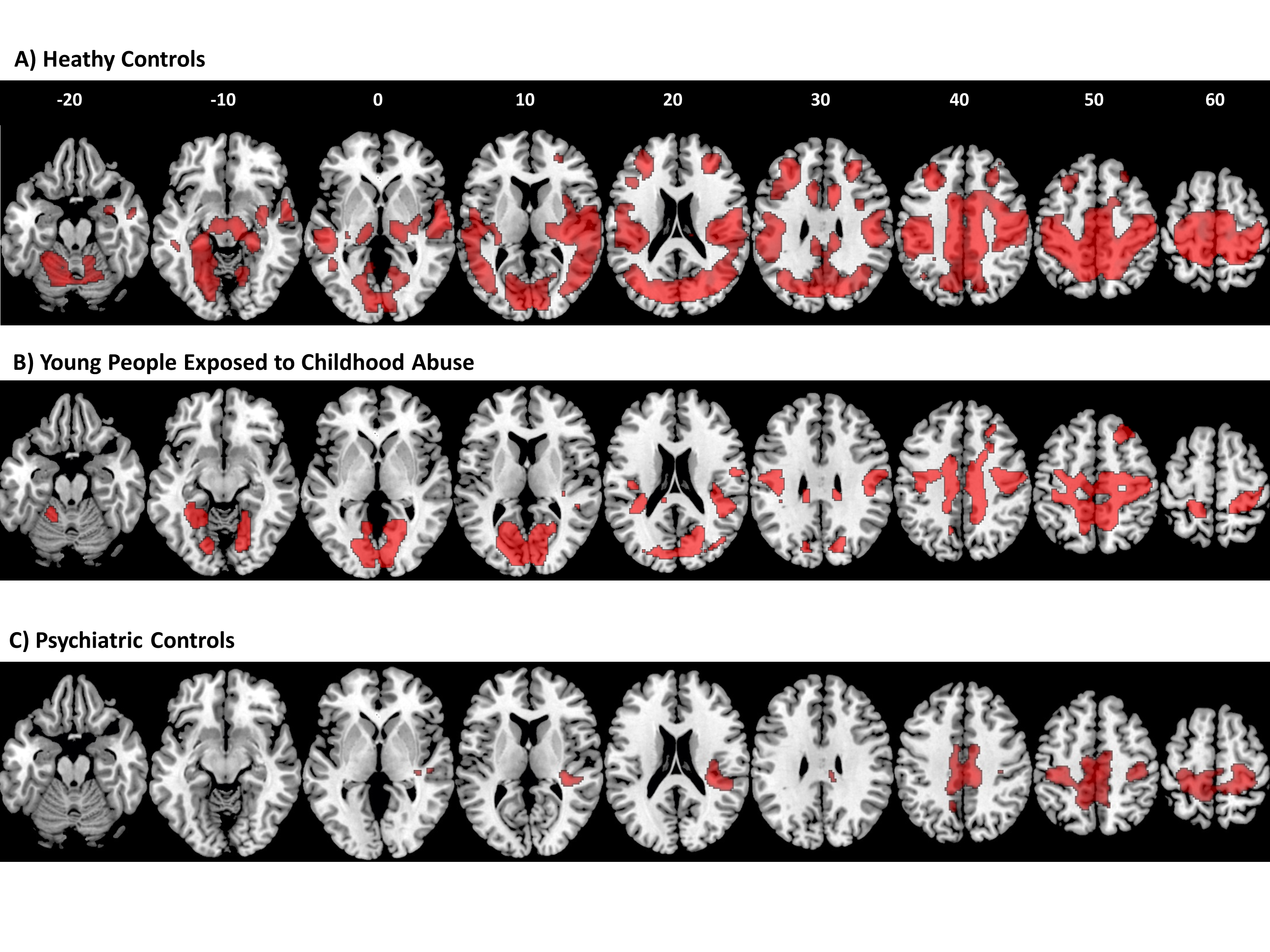

Supplement: S4 Fig — Axial sections of activation during 2s, 5s and 8s delays for 27 healthy controls, 21 young people exposed to childhood abuse and 19 psychiatric controls at FWE-corrected cluster-level threshold p < 0.05. Axial slices are marked with the z coordinate as distance in millimetres from the anterior–posterior commissure. The right side of the image corresponds to the right side of the brain. (TIF) [file pone.0165547.s004.tif]

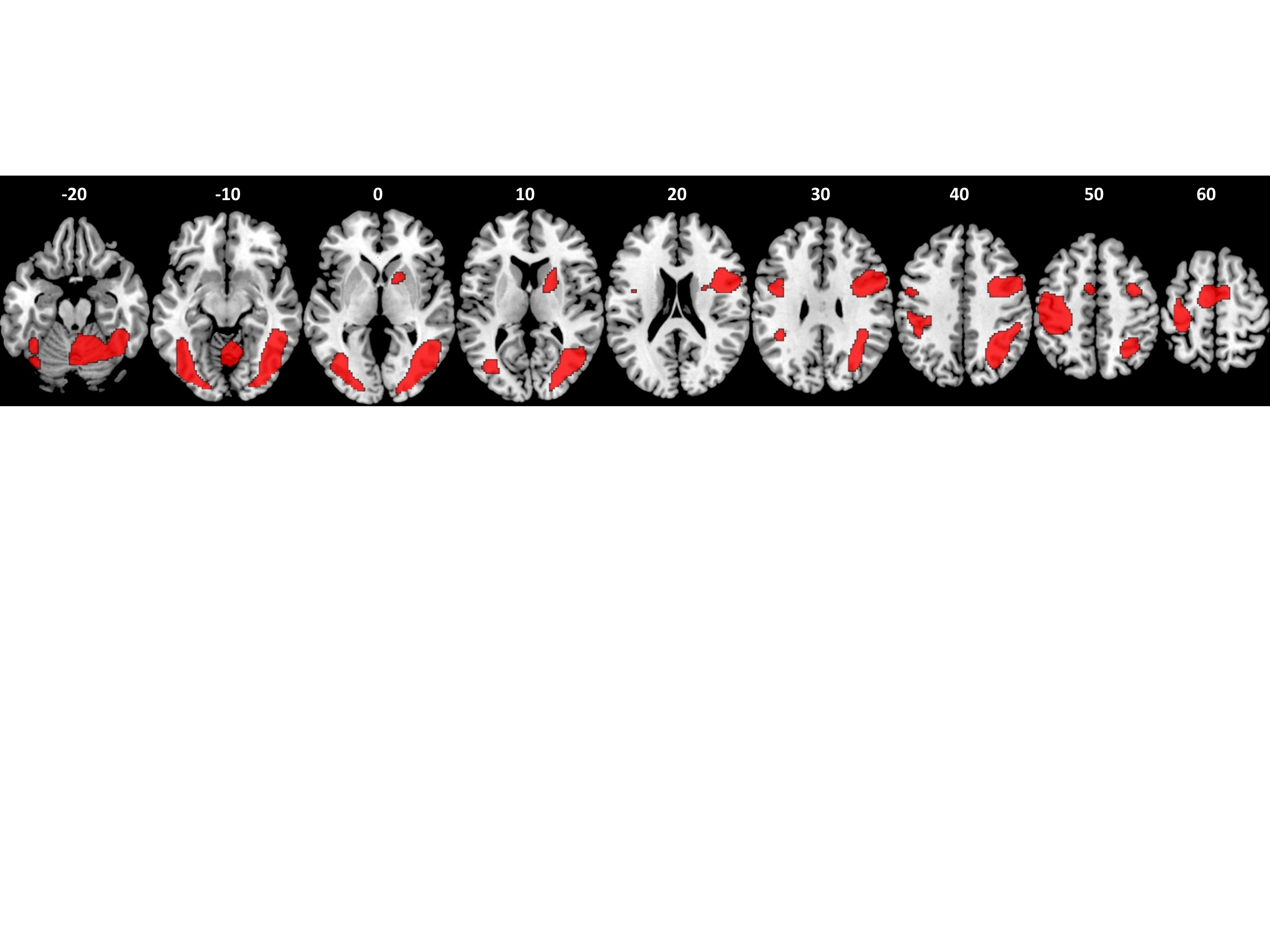

Supplement: S5 Fig — Axial sections showing main effect of delay on brain activation during sustained attention across 27 healthy controls, 21 young people exposed to childhood abuse and 19 psychiatric controls, as revealed by F test, p < 0.05 FWE-corrected at the cluster-level. Axial slices are marked with the z coordinate as distance in millimetres from the anterior–posterior commissure. The right side of the image corresponds to the right side of the brain. (TIF) [file pone.0165547.s005.tif]
