# Supplementary material for: Neurofunctional Abnormalities during Sustained Attention in Severe Childhood Abuse
Source: PLoS One. 2016 Nov 10;11(11):e0165547. doi: 10.1371/journal.pone.0165547 (PMC5104469; doi:10.1371/journal.pone.0165547)
Supplement: S1 Table — (DOCX) [file pone.0165547.s006.docx]

**S1 Table. Performance Measures for the Sustained Attention Task during 0.5s Delay for 21 Young People Exposed to Childhood Abuse, 19 Psychiatric Controls and 27 Healthy Controls.**

|  | **Healthy Controls (N=27)** | |  | **Childhood Abuse**  **(N=21)** | |  | **Psychiatric Controls (N= 19)** | |  | **Analysis** | | |
| --- | --- | --- | --- | --- | --- | --- | --- | --- | --- | --- | --- | --- |
|  | **Mean** | **SD** |  | **Mean** | **SD** |  | **Mean** | **SD** |  | **F(2, 64)** | ***p*(corr.)** | **Group Comparisons** |
| MRT | 320 | 81 |  | 343 | 86 |  | 304 | 67 |  | 1.24 | 0.30 | - |
| SDintrasubject | 78 | 32 |  | 115 | 35 |  | 100 | 19 |  | 9.38 | <0.001 | CA, PC > HC |
| Omission errors | 1.00 | 1.44 |  | 8.33 | 15.5 |  | 3.84 | 7.73 |  | 3.45 | 0.04 | CA > HC |
| Premature errors | 9.70 | 19.8 |  | 20.5 | 16.7 |  | 10.5 | 9.67 |  | 2.80 | 0.07 | - |

MRT=mean reaction time (in ms); SDintrasubject=intrasubject variability of response of reaction time (in ms); corr=Bonferroni corrected; CA=childhood abuse; HC=healthy control; PC=psychiatric control
